# The Homeopaths at It Again

**Published:** 1893-03

**Authors:** 


### THE HOMEOPATHS AT IT AGAIN.

The homeopaths are at their tricks again. Representative Finlay, of Galveston, has introduced into the Legislature, now sitting, a resolution to require the Board of Regents of the University of Texas, to appoint one eclectic and one homeopath on the Faculty of the Medical Department (Texas Medical College).

There is reason to fear they will do it. The homeopaths claim that the laws of Texas say that "no discrimination shall be made between schools of medicine,"—and they are now appealing to the law.

The JOURNAL does not recognize any such thing as "schools of medicine;" there is but one "school" of medicine, as there is but one "Church;" but there are several "denominations," and there are several "pathies." Should the Legislature recognize any "pathy" as a "school" of medicine, and comply with the absurd demands of these fanatics, they must go still further, and recognize them all. Where can the line be drawn between *homeopathy* and *hydropathy—electropathy*, or *Christian science?* If an eclectic—who, as the name implies, is neither one thing nor another, but a kind of nondescript, who will give anything for any kind of disease, or a homeopath, be appointed,—who is only a homeopath in name (and for revenue), and who will, when he gets in a tight place—as the JOURNAL has often said—throw away their principle (?) of *similia similibus*, and administer "strong medicine," in "allopathic" doses,—what excuse can be given for not going the whole swine, and putting on the staff a "Christian scientist"? The "Christian scientists" are at least consistent.

There is no "allopathy;" that is a name spitefully bestowed upon the practitioners of regular medlcine by these Hahne-*maniacs;* and people associate the term "allopathy" with large doses of nauseous drugs. It is high time they were undeceived on this point; but one had as well whisper to the wind as to talk to the average Texas Legislator on the subject: their *sympathies* are with the homeopaths, and they are going to recognize them; mark the prediction!

But what sort of a school will be the result? The only difference between *any* of the so-called "schools," is the *Materia Medica*, and *Practice*. All are taught (if taught) all the other branches precisely alike. A homeopath or an eclectic, or any other kind of irregular, is taught the same kind of anatomy; it is a fixed thing; there are no varieties; there is no such thing as homeopathic "anatomy," nor homeopathic "surgery," nor "obstetrics," nor "chemistry," nor "hygiene;" they are the same in all "schools." Where, then, would be the *sense* in appointing a *homeopath* to teach either of these branches? The only branches in which there is any distinction between homeopaths and regular physicians, as stated, are, "Practice" and "Therapeutics;" their respective modes of practice and their *Materia Medica;*

these are the two branches which give character and name to a school; and were a homeopath appointed to the chair of Practice, or the chair of Therapeutics and Materia Medica, in our University Medical College, it would become at once a homeopathic college, and no *physician*—and when we say physician, we mean a physician, and not a homeopath, or other mongrel,—could consistently hold either of the other chairs;—hold a professorship in a homeopathic college!

Should the Legislature, therefore, be hoodwinked by the specious pleas of these persevering irregulars, and induced, through mistaken notions of right or justice, or through their sympathies for the little dog in the fight, to appoint one of their ilk on the Faculty, it will make a sad mess of it; if to the chair of Practice, they thus convert it into a homeopathic institution, and as it will drive the others out, it will be tantamount to turning the College over to them altogether. If one be appointed to any other chair, it would be but the name of the thing, and would doubtless have no other effect than to break up the school; as no student who has studied under a reputable preceptor—a regular physician,—could be expected to want a diploma from a mixed school,—it would be neither one thing nor another.

The alternative, in our opinion, is to appoint all or none of the professors from these "sects;" to make it either a *homeopathic* college, or let it remain as it is,—a *Medical* college.

